# Impact of initial postweaning feed intake on weanling piglet metabolism, gut health, and immunity

**DOI:** 10.1093/jas/skaf099

**Published:** 2025-03-31

**Authors:** Lluís Fabà, Susana M Martín-Orúe, Tetske G Hulshof, José Francisco Pérez, Michael O Wellington, Hubèrt M J Van Hees

**Affiliations:** Trouw Nutrition R&D, Swine Research Centre, Boxmeer, The Netherlands; Animal Nutrition and Welfare Service, Department of Animal and Food Sciences, Universitat Autònoma de Barcelona, Bellaterra, Spain; Trouw Nutrition R&D, Swine Research Centre, Boxmeer, The Netherlands; Animal Nutrition and Welfare Service, Department of Animal and Food Sciences, Universitat Autònoma de Barcelona, Bellaterra, Spain; Animal Nutrition and Welfare Service, Department of Animal and Food Sciences, Universitat Autònoma de Barcelona, Bellaterra, Spain; Trouw Nutrition R&D, Swine Research Centre, Boxmeer, The Netherlands; Ghent University, Faculty of Veterinary Medicine, Department of Veterinary and Biosciences, Merelbeke, Belgium

**Keywords:** feed intake, gut health, metabolic stress, immunity, gut-brain-axis, nursery pig

## Abstract

Low feed intake (**FI**) in weanling pigs can be hypothesized as both a cause and consequence of intestinal disturbances and metabolic stress. We explored the associations between individual daily FI patterns, metabolic status, and intestinal physiology. Female pigs (*n* = 24) were selected based on high or low cumulative FI between d1 and d3 relative to weaning (d0) from 12 pens equipped with electronic feeding stations at 1-wk after weaning for dissection and sampling. Four classes of pigs were created with pigs that started with a high or low FI (d1 to d3) and continued with a high or low FI (d4 to d6) (HH, HL, LH, and LL, respectively; *n* = 6) for data analysis. In plasma, HL pigs showed higher plasma glutamate dehydrogenase than LL pigs (*P* < 0.05). A low FI d1 to d3 increased plasma creatinine and lactate dehydrogenase, and reduced insulin-like growth factor (IGF-I), gastrointestinal organ weights, and jejunal villus surface area at 1 wk after weaning (*P* < 0.05). However, low FI d4 to d6 increased plasma haptoglobin, PigMAP, bile acids, and bilirubin levels and reduced jejunal villus length (*P* < 0.05). In jejunum tissue, HH pigs had the highest jejunal upregulated IGF-I receptor and a reduced local inflammatory gene expression when compared to HL pigs (MyD88), and similarly, when compared to all classes (FAXDC2). For the main effects, pigs classified as high FI d1 to d3 had upregulated immune systems including IL-6, TGFB1, TLR2, and TLR4 genes compared to low FI d1 to d3 pigs (*P* < 0.05). In a multivariate model, variance in average daily gain (*R*^2^ = 0.82) was mostly explained by positive correlations with FI d1 to d3, jejunal morphometrics, and plasma IGF-I, while negatively explained by histamine in digesta, and creatinine, PigMAP, triglycerides, and haptoglobin in plasma. In conclusion, pigs transitioning from high to a low FI showed distinct metabolic alterations and a subtle local inflammation masked by the vigorous local immune response in pigs with initial (d1 to d3) high FI. Pigs with an initial low FI had a fasting-like metabolic state, indicated by hepatic alterations pointing at shifting protein metabolism into energy production. Altogether, FI during the initial days postweaning significantly impacts pig growth, immunity, and metabolism, with a sustained low intake (i.e., up to 6 d) triggering a systemic inflammatory response.

## Teaser

Early postweaning feed intake patterns shape piglet growth, intestinal health, and immune function, highlighting the need for strategic nutritional planning.

## Introduction

The use of individual feed intake (**FI**) measurements in nursery pig research is still scarce; however, such data could help researchers to understand the adaptation to solid feed following weaning. Recent work from our group demonstrated that pigs with high voluntary FI between d1 and d3 after weaning showed an improved FI and growth towards the end of the nursery (Fabà et al., 2024a, 2024b). An explanation for such a carryover benefit may be the cause and consequence of improved gut health since high FI pigs were observed to have an increased villus length and surface area in jejunum and a reduced biogenic amine in the small intestine (**SI**) digesta 1-wk after weaning compared to their low FI counterparts (Fabà et al., 2024a). There is evidence that the stress and inflammation associated with the weaning event concur with reduced FI and growth performance in weanling pigs (Pluske et al., 1996; Pié et al., 2004; Lallès et al., 2007; Moeser et al., 2007). Similarly, a reduced FI is expected to cause inflammation. In a recent study, 35-d-old pigs exhibited an upregulated inflammatory response in the jejunum after 7 d of high-intensity feed restriction (20% compared to 100% free access), but not after 3 d (Wang et al., 2025). Engelsman et al. (2023), reported that individually housed pigs with high FI between weaning and d4 had an improved growth performance as well as an increased jejunal villus surface area at the end of the nursery. The paradox, as we see it, is that high-performing pigs had increased inflammatory markers in serum and a higher risk of diarrhea compared to their low-FI counterparts (Engelsman et al., 2023). Thus, investigations are warranted to further understand the importance of distinct feeding patterns immediately after weaning on later life performance and health.

To date, it is unknown how important the FI pattern along the first days after weaning is for gastrointestinal physiology and risk for disturbances. For instance, whether d1 is as important as d2, d3, etc. or whether an abrupt increase in FI may be more critical than a gradual increase. It could be hypothesized that both, one) a poor FI during the first few days after weaning, and two) a sudden increase in FI after a few days of poor FI, may have negative consequences for intestinal functions and physiology. It can be speculated that the weaning event can generate a circular response in some pigs where low initial FI leads to poorer gut health (Pluske et al. 1996, 1997), disrupted endocrine system (Carroll et al., 1998), and an increased intestinal immune response (Pié et al., 2004), which in turn can lead to reduced FI and metabolic dysfunction (Jayaraman and Nyachoti, 2017; Southey et al., 2021). The recovery process, such as villi reconstruction and recovery of the mucosal barrier and digestive functions, is further hampered by the presence of unabsorbed nutrients. Among these nutrients, protein is the main risk factor for intestinal dysbiosis (Kim et al., 2012; Pieper et al., 2012; Zhang et al., 2020; Li et al., 2022). In the weanling pig, the disrupted brush border lacks detoxifying enzymes such as intestinal alkaline phosphatase (**ALPi**) which further increases the risk of an infectious disease (Lackeyram et al., 2010). In addition, an abrupt increase in FI following a fasting period triggers insulin spikes by the so-called refeeding syndrome which results in edema formation, downshifts in metabolic activity, and associated shedding of minerals in urine (Van Kempen et al., 2023). As mentioned before, most research has not focused on individual FI progression after weaning. Hence, connecting individual FI daily measurements to key intestinal and physiological biomarkers could help understand how pigs adapt to solid feed following weaning and what consequences for intestinal function and integrity as well as growth performance it has.

We hypothesize that immune and metabolic indicators can identify a negative response in pigs transitioning their initial FI between low and high patterns, or vice versa. Consistently low FI during the first week postweaning, or transitioning from high to low intake midweek, is expected to increase energy metabolic efforts, hepatic dysfunction, and inflammatory markers both locally and systemically by the end of the week. The objective of the present study was to characterize associations between daily FI during the first days after weaning and the effects on key biomarkers of metabolism, intestinal physiology, and immunity of piglets in the immediate postweaning period.

## Material and Methods

### Ethical approval

The experimental protocol was approved and followed the Central Committee Animal Experimentation (CCD, The Hague, The Netherlands) approval with animal use protocol # AVD2040020184665.

### Animals, housing, and experimental design

The present study used data originating from a larger experiment and details of the methodology can be found there (Fabà et al., 2024a). Briefly, the original study included 144 mixed-sex pigs (Hypor Libra × Hypor Maxter, Hendrix Genetics B.V., Boxmeer, The Netherlands) in a 40-d study at the Trouw Nutrition R&D Swine Research Centre (St. Anthonis, The Netherlands). Pigs were weaned at 24 ± 2 d of age [7.16 ± 0.80 kg body weight (**BW**)] and allocated to 2 rooms with 6 pens/room and 12 pigs/pen. The allocation was based on BW at weaning (BW, d0), litter origin, and sex with 5 males and 7 females per pen. Each pen (3.0 m × 1.67 m) was equipped with an electronic feeding station (**EFS**, Schauer Agrotronic, GmbH, Austria) having 15 cm single feeder space. The EFSs were activated by the pig’s ear tag Radio-Frequency IDentification (RFID, MS Tag Round HDX STF-YELOPPRPB1, MS Schippers B.V., Hapert, The Netherlands) enabling monitoring of individual FI and feeding pattern. Room lighting was on between 0600 and 2200 hours throughout the study and room temperature was initially set at 30 °C at weaning and gradually reduced to 25 °C on d28 and maintained until the end of the nursery. Two experimental diets, that met or exceeded the nutrient requirement of weanling pigs (NRC, 2012), were offered ad libitum during d0-15 and d15-40, respectively (Fabà et al., 2024a). Water was available ad libitum via drinking nipples with one nipple per pen.

For the present study, two female pen mates were selected for euthanasia and tissue sampling at 1-wk postweaning (*n* = 24 in total, 8.0 ± 1 kg BW at day 6). Selection was based on FI during d1 to d3 with one female pig having the highest FI d1 to d3 and another female having the lowest FI d1 to d3 within a pen. The FI d1 to d3 criterion was chosen based on literature (Le Dividich and Herpin, 1994; Bruininx et al., 2001) as having significant effects on intestinal health and performance (Spreeuwenberg et al., 2001; Engelsmann et al., 2023). Females were selected for having more adverse responses in intestinal gut health relative to males as caused by weaning stress (Pluske et al., 2019). Hence, only females were selected to reduce variation and minimize sample size. The two groups of pigs with extreme FI, which were equally distributed between pens, were further divided in two classes regardless of pen. Therefore, four classes were made by dividing pigs with high or low FI between d1 and d3 (*n* = 12) and dividing those pigs again by classifying them based on high or low FI between d4 and d6 (HH, HL, LH, and LL, respectively; *n* = 6 per class).

### FI and growth performance

The EFS recorded feed disappearance and the start and end time of each visit per pig. Data included the daily number of visits to the trough, time per visit, and amount consumed per visit from d1 after weaning onwards. The d6 is incomplete and approximately only half a day is included due to pigs being processed for sampling. To avoid differences due to dissection order and time, when reporting the FI of d6, we calculated the FI for the last 24h prior to euthanasia. This is redundant and only done to allow d6 FI comparisons and correlations between other variables. When reporting and using the average daily feed intake (**ADFI**) from d1 to d6, only the feed disappearance of d6 until euthanasia of individual pigs is included. Individual BW was determined on d0 and d6. The average daily gain (**ADG**) and gain-to-feed ratio (as d0 to d6 ADG: d0 to d6 ADFI) were calculated for the corresponding period. The NE maintenance requirements (in terms of the amount of FI) were calculated for every pig using 750 kJ NE/kg^0.6^ (Everts, 2015), individual BW at weaning, and NE concentration of the feed (10.6 MJ NE/kg). For the four FI classes, the proportion of pigs consuming feed above or below maintenance was calculated.

### Sample collection

The order of pigs selected for euthanasia was by pen, which means that both pigs, i.e., the highest and lowest FI d1 to d3 for each pen, were sampled consecutively. In brief, the 24 female pigs selected for tissue sampling were sedated using a mixture of Zoletil (250 mg zolazepam and 250 mg tiletamine; VIRBAC, Carros, France) and 20 mL Sedanum (20 mg xylazine / mL; Dechra Pharmaceuticals, Northwich, United Kingdom) at 1 ml per 10 kg BW and, thereafter, humanely euthanized by intracardiac injection with 40% barbiturate pentobarbital (390 mg pentobarbital sodium and 50 mg phenytoin sodium per mL, Euthasol, Virbac, Carros, France). After opening the abdominal cavity, a blood sample was collected from the portal vein to collect plasma. Blood was collected in 5 mL tubes (S-Monovette Lithium heparin gel+ LH, SARSTEDT AG & Co. KG, Germany) centrifuged 10 min at 2,000 × *g* at 4 °C, then the supernatant plasma was stored at −20 °C until further analysis. After taking the blood sample, the abdominal organs were removed. The full organ weights were recorded, and contents were collected for further analysis, after which the empty organ weights were measured. The empty organ weights and content weights by section were expressed in g/kg BW at euthanasia.

A homogeneous 50 g content sample from pooled SI sections (duodenum, jejunum, and ileum content) was collected and snap-frozen on dry ice and stored at −20 °C until further analysis. One 3 cm tissue sample from jejunum was obtained from 30 cm caudal to the pylorus and fixed in a cassette including 4% buffered formaldehyde for histomorphometry measured by the laboratory GD animal health (Royal GD, Deventer, The Netherlands). Immediately next to this jejunal tissue location, one sterile 4 mm biopsy punch (MILTEX 33-34 Biopsy Punch, Integra LifeSciences, Princeton, NJ, USA) was collected after rinsing with phosphate-buffered saline. The sample included all intestinal wall layers and was placed in a sterile cryotube with RNAlater (Life Technologies, Carlsbad, CA, USA) and kept at 4 °C until later analysis for gene expression. From the same jejunal location described above and another location from ileum, 10 cm anterior from the ileocecal valve, a 5 cm length section per tissue was collected after rinsing with phosphate-buffered saline and placed into −70 °C until later processing for ALPi.

### Experimental procedures

#### Metabolites, enzymes, and biomarkers in plasma

Portal vein plasma samples were submitted to the veterinary laboratory of Royal GD (Deventer, The Netherlands). Clinical–chemical parameters were assessed using clinical–chemical analyzer and results were evaluated according to the quality control procedures of the laboratory (meeting NEN-EN-ISO 9001:2015 requirements). Colorimetric methods were used to analyze Ca, P (ammonium-molybdate method), Mg, total protein (Biuret method), albumin (Bromocresol Green method), and total bilirubin concentrations (all test kits from Beckman Coulter; Brea, CA, US). Enzymatic methods were used to analyze plasma concentrations of urea (urease method; test kit Beckman Coulter; Brea, CA, US), creatinine (test kit Beckmann Coulter; Brea, CA, US), and non-esterified fatty acids (**NEFA**) (Wako NEFA-HR(2) test kit; FUJFILM Wako Chemicals Europe GmbH; Neuss, Germany). Creatine phosphokinase (CPK) concentrations in plasma were analyzed using enzymatic methods according to the International Federation of Clinical Chemistry (**IFCC**) reference procedures (test kit Beckmann Coulter; Brea, CA, US). Glutamate dehydrogenase (GLDH) concentrations in plasma were analyzed using an enzymatic method according to the DGKC (GLDH3 test kit, Roche Diagnostics GmbH; Basilea, Switzerland). Pig Major Acute-phase Protein (PigMAP) concentrations were analyzed using a turbidimetric assay (Turbovet pig-MAP test kit, Acuvet Biotech, Spain). All these parameters were analyzed on a DxC 700 AU Chemistry Analyzer (Beckman Coulter; Brea, CA, US). The remaining parameters were analyzed on a UniCel DxC 600 Synchron Clinical System (Beckman Coulter; Brea, CA, US). Plasma concentrations of aspartate aminotransferase (AST), alkaline phosphatase (ALP), and gamma glutamyl transpeptidase were analyzed using enzymatic methods according to the IFCC reference procedures and concentrations of alanine aminotransferase (ALT) and lactate dehydrogenase (LDH) were analyzed using enzymatic methods according to modified IFCC reference procedures (all test kits were from Human Diagnostics, Beckman Coulter; Brea, CA, USA). Haptoglobin concentrations were analyzed using a colorimetric method (“PHASE” Haptoglobin Assay; Tridelta Development Ltd; Kildare, Ireland). All methods used kits which were used according to manufacturer instructions.

The plasma samples were also submitted to the Veterinary Clinical Biochemistry Service from Autonomous University of Barcelona (Bellaterra, Spain). Triglycerides were analyzed via the enzymatic colorimetric procedure with glycerol phosphate oxidase and glucose via the hexokinase method. For both, OSR (Olympus System Reagent, Beckman Coulter, Brea, CA, USA) was used. Bile acids were analyzed via the enzymatic colorimetric method with 3 alpha-hydroxysteroid dehydrogenase. Triglycerides, glucose, and bile acids were evaluated according to the quality control procedures of the laboratory (meeting NEN-EN-ISO 9001:2015 requirements) and were analyzed using an Olympus AU480 analyzer (Beckman Coulter; Brea, CA, USA).

### Hormones in plasma

The plasma samples, that were submitted to the Veterinary Clinical Biochemistry Service from Autonomous University of Barcelona (Bellaterra, Spain), were also analyzed for hormones. Samples were analyzed for insulin-like growth factor-I (IGF-I) concentrations using an IGF-I ELISA kit (Ref. E20, Mediagnost, Reutlingen, Germany), cortisol using a cortisol ELISA kit from DRG Instruments (EIA-1887, DRG Instruments, Marburg, Germany), and serotonin using an ELISA Kit (Ref: ADI-900-175, ENZO Life Sciences, Switzerland). Analyses were conducted according to the kit’s manual. Plates were read at 450 nm using EMS Reader MF V.2.9-0 from Labsystems (Vantaa, Finland).

### Alkaline phosphatase in intestinal tissue

The analysis was conducted at the veterinary laboratory of Royal GD (Deventer, The Netherlands) using a modified methodology from Lackeyram et al. (2010). The jejunal and ileal tissue samples preserved at −70 °C were subsampled as 1.0-1.2 g from the middle tissue, including all intestinal wall layers, with a scalpel and a Petri dish. Next, the approximately 1.0 g sample was thawed in an ice-cold homogenizing buffer (50 mmol/L d-mannitol and 0.1 mmol/L PMSF at pH 7.4) at a ratio of 20 mL homogenizing buffer/g frozen intestinal tissue sample and homogenized using a polytron homogenizer and the plastic dispersion tools (Ultra Turrax, Breisgau, Germany). The resulting homogenate samples were centrifuged for 10 min at 2,000 × *g* at 4 °C. After that, 5 ml supernatant was transferred into a new tube and analyzed using a DxC 700 AU Chemistry Analyzer (Beckman Coulter, Brea, CA, USA). In each supernatant, the concentration of ALPi was analyzed according to the IFCC reference procedures using an Alkaline Phosphatase test kit from Beckman Coulter and expressed as ALPi, IU/g tissue.

### Jejunal histomorphometry measurement

Morphometric measurements were conducted by a pathologist board certified veterinarian at the laboratory GD animal health (Deventer, The Netherlands) and details are described elsewhere (Fabà et al., 2024a). For the present study, among all morphometric measurements, villi surface area, villus length, and crypt depth were used because of their significant associations with FI as previously reported (Fabà et al., 2024a).

### Biogenic amines analysis

The content of biogenic amines including cadaverine, histamine, putrescine, spermidine, and spermine in SI pooled content were analyzed by HPLC on a BioRad Aminex HPX-87H as described elsewhere (Fabà et al., 2024a).

### Analysis of crude protein in small intestinal digesta

The SI content samples were dried at 70 °C to constant weight in a Memmert oven (Memmert GmbH, Schwabach, Germany) to obtain the dry matter concentration (Masterlab, Boxmeer, The Netherlands). The dried samples were milled (Retsch ultra centrifugal mill) using a 0.75 mm sieve. Crude protein (**CP**) was analyzed as nitrogen by the combustion method (method 990.03; LECO FP 528 MI, USA) using the LECO Nitrogen analyzer, and CP was calculated as nitrogen × 6.25.

#### Gene expression analysis

From 30 mg of frozen jejunum tissue, the RNA was obtained using the Maxwell RSC simplyRNA Tissue Kit (Promega Corporation, Madison, WI, US) in a Maxwell RSC instrument (Promega Corporation) following the manufacturer’s instruction. The quality and quantity of RNA were assessed with a NanoDropND-1000 spectrophotometer (NanoDrop products, Wilmington, DE, USA) whereas the RNA was via Agilent Bioanalyzer-2100 equipment (Agilent Technologies, Santa Clara, CA, USA). Reverse transcription of approximately 1 µg of total RNA to single-stranded cDNA in a final volume of 20 μL was performed using a High-Capacity cDNA Reverse Transcription kit (Applied Biosystems, Foster City, CA) and random hexamer primers. The thermal cycler conditions applied were 25 °C for 10 min, 37 °C for 120 min; 85 °C for 5 min, and 4 °C hold.

Gene expression was analyzed by high-throughput qPCR using a 96.96 Dynamic Array™ IFC for Gene Expression (Standard Biotools Inc., South San Francisco, CA, US) in a Biomark™ HD (Standard Biotools Inc., South San Francisco, CA, US) system. The samples were processed per duplicate at 1/20 dilution for the mRNA quantification of 47 selected genes (43 target genes and 4 reference genes) based on previous results (Villagómez-Estrada et al., 2022). The genes were selected based on literature involved in intestinal health and grouped according to their main physiological function: 1) barrier function genes such as mucins (*MUC2*, and *MUC13*), zonula occludens (*ZO1*), trefoil factor 3, and occludin (*OCLN*); 2) the immune and inflammatory functions such as pattern recognition receptors, cytokines, chemokines, and stress proteins [toll-like receptor (*TLR2*, *TLR4*); TLR adaptor Myeloid Differentiation Primary Response Protein (*MyD88*); Nuclear Factor Kappa-betta (*NFK*-β); S100 Calcium (and zinc) Binding Protein A9 (*S100A9*); interleukin (*IL1β, IL-6, IL8, IL-10*); interferon gamma receptor; tumor necrosis factor alpha (*TNF-α*); transforming growth factor beta 1 (*TGF-β1*); chemokine ligand; heat shock protein; peroxisome proliferator activated receptor alpha; fatty acid hydroxylase domain containing 2 (*FAXDC2*), and guanylate binding protein]; 3) antioxidant enzymes genes such as glutathione peroxidase, superoxide dismutase (*SOD2*); 4) digestive enzyme and hormone genes involved in the digestion and metabolism processes [intestinal alkaline phosphatase (*ALPi*); sucrase-isomaltase; d-amino-acid oxidase; histamine N-methyltransferase (*HNMT*); alanyl aminopeptidase membrane (*ANPEP*); indoleamine 2,3-dioxygenase (*IDO1*); cholecystokinin (*CCK*); IGF-I receptor (*IGF1R*); and E3 Ubiquitin-Protein Ligase]; 5) nutrient transport coding genes [(solute carrier family (*SLC5A1*, *SLC7A8*, *SLC13A1, SLC15A1, SLC16A1*, and *SLC39A4*), Proline-Rich AKT1 Substrate 1 (*AKT1S1*)]; and 6) stress response genes [corticotropin releasing hormone receptor (*CRHR1*); and hydroxysteroid (11-beta) dehydrogenase (*HSD11β1*)].

Data collection and quality control were performed in the software Fluidigm Real-Time PCR Analysis (Standard Biotools Inc., South San Francisco, CA, US) (v4.8.1). Then, the software DAG Expression (v1.0.5.6) (Ballester et al., 2013) was utilized for the analysis. The relative standard curve method was applied to account for the gene-specific efficiency of the qPCR, generating quantity values. In addition, normalization of these gene expression levels was performed using four reference genes (*ACTB, B2M, GAPDH*, and *TBP*), generating the normalized quantity values. Gene expression data were analyzed by applying the 2−ΔΔCt method for relative quantification and using the sample with the lowest expression as a calibrator.

### Statistical analysis

The normality of data was checked by visual assessment of residual plots and PROC UNIVARIATE (SAS Inst. Inc., Cary, NC, USA). The four classes for pigs (HH, HL, LH, and LL) which started high or low FI (d1 to d3) based on the selection criteria and then continued as high or low FI (d4 to d6) were studied in a 2 × 2 factorial. Dissection data were analyzed using PROC GLIMMIX and included the fixed effects of FI d1 to d3 class and FI d4 to d6 class (high or low) and their interaction, and pen as the random effect. For the daily FI, a similar model as for the dissection data was created including time (day after weaning) and the interaction with the FI classes as fixed effects and the intercept with animal as random effect to account for the repeated measure. The PROC CORR was used to determining Pearson correlations and construct correlation heatmaps between variables of daily FI and biomarkers. In addition, the biomarkers were correlated to SI weight, pancreas weight, jejunum villus length, and villus surface area to support the discussion of results. PROC REG was used to describe linear relationships and the coefficient of determination between variables. In the PROC GLIMMIX procedure, normally distributed data were analyzed with dist=gaussian and link=identity and non-normal data (i.e., biogenic amines, gene expression, plasma biomarkers that did not distribute normally) were analyzed with dist=gamma and link=log.

Eventually, all data were combined to identify the most important associations using multivariate models to explain the development of FI and growth during the first week after weaning. PROC PLS was used to create partial least squares (**PLS**) multivariate models to explain: 1) variance of daily FI and 2) variance of ADG d0 to d6. The final PLS models included variables selected based on preliminary screening via significant Pearson correlations. Once the first models were created, further selection was done keeping the most important variables based on the importance plot from PROC PLS in SAS.

The mean separation was done using the PDIFF option and adjusted using the Tukey–Kramer test. The experimental unit was the individual pig. The *P*-values < 0.050 were considered significant for all data, and *P* < 0.100 were considered trends.

## Results

### Feeding patterns

**Figure 1.**
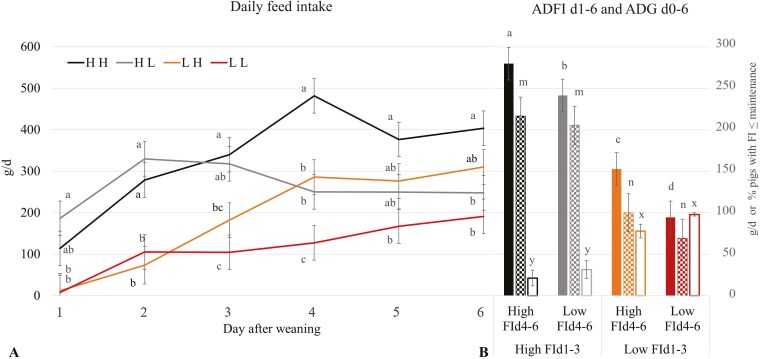
(A) Daily FI and (B) ADFI (solid fill) for d1 till d6, ADG (dotted fill) between d0 (weaning) and 6, and proportion of pigs with daily FI below maintenance [Calculated using weaning BW, 750 kJ NE/kg BW^0.6^ (Everts, 2015), and the dietary net energy concentration (10.6 MJ/kg)] (framed) for pigs separated in four classes (*n* = 6) based on FI pattern. Pigs with high or low feed intake for d1 till d3 (FId1-3) which was followed by either a high or low FI for d4 till d6 (FId4-6) after weaning (HH, HL, LH, and LL, respectively). Panel A: ^a,b,c^LSMEANS within a day without a common superscript differ at *P* < 0.05. Panel B: ^a,b,c^LSMEANS for ADFI, ^m,n^for ADG, and ^x,y^for proportion of pigs with FI below maintenance requirements between FI classes without a common superscript differ at *P* < 0.05.

The daily FI of the four classes of pigs (HH, HL, LH, and LL) are shown in Fig. 1A. The ADFI d1 to d6 was intentionally different between classes (*P* < 0.05) with HH being the highest (280 g/d), followed by HL (242 g/d), which in turn was higher than LH (153 g/d), and subsequently LL (95 g/d) as the lowest (*P* < 0.05; Fig. 1B). On average, the 24 pigs in the study showed a progression in FI, with day-to-day relative increases of 60%, 14%, 18%, -7% for d1 to d2, d2 to d3, d3 to d4, and d4 to d5, respectively. FI was highly variable and daily coefficient of variation were 113%, 75%, 53%, 64%, and 42% for d1 to d5, respectively. The LH and LL maintained a steady increase in FI while HL reduced FI by ~20% during d4 to d6 relative to d3 and HH reduced FI by ~19% from d5-6 FI to d4 (both *P* < 0.05). The ADG d0 to d6 was only different between the FI d1 to d3 classes resulting in HH and HL having on average a 60% higher gain than LH and LL (211 vs 84.7 g/d, *P* < 0.05; Fig. 1B) while gain-to-feed ratios were not significantly different (*P* > 0.05) being 0.78, 0.85, 0.66, and 0.73 for HH, HL, LH, and LL, respectively. In addition, the proportion of days with pigs having a FI below their maintenance requirements was higher for low (93%) vs. high FI d1 to d3 (26%) and low (83%) vs. high (49%) FI d4 to d6 (*P* < 0.03 for both) without an interaction between classes (Fig. 1B). The higher ADFI of the HH and HL pigs was explained by a higher number of visits on d1 (*P* < 0.05) and 2 (*P* < 0.10; Fig. S1) and more time spent per visit on d1-4 (*P* < 0.05; Fig. S2) compared to the LH and LL pigs.

### Metabolic response

The responding biomarkers measured in portal vein plasma and evaluated for describing pig’s physiological and metabolic status between the FI classes are reported in Table 1 and the non-responding biomarkers are reported in Table S1. The only significant interaction between the FI d1 to d3 and FI d4 to d6 classes was for GLDH (*P* = 0.029), with HL pigs showing higher values than LL pigs with HH and LH being intermediate and not different (Table 1). Creatinine showed an opposite tendency for an interaction (*P* = 0.052) between FI classes with LL being higher than HL and HH and LH being intermediate and not different (Table 1). As main effect, a high FI d1 to d3 resulted in increased IGF1 (*P* < 0.001) and reduced creatinine (*P* = 0.015), urea (*P* = 0.050), and LDH (*P* = 0.044) while tended to reduce triglycerides (*P* = 0.081), PigMAP (*P* = 0.052) and cortisol (*P* = 0.100) in plasma of pigs 1-wk after weaning compared to low FI d1 to d3. For FI d4 to d6, a high FI resulted in reduced haptoglobin (*P* = 0.006), PigMAP (*P* = 0.021), bilirubin (*P* = 0.022), and bile acids (*P* = 0.049) and tended to increase albumin (*P* = 0.093) and ALP (*P* = 0.100) compared to low FI d4 to 6. Finally, there were no significant differences between the high or low FI classes during d1 to d3 or d4 to d6 (*P* > 0.10) for the other biomarkers, i.e., ALT, AST, CPK, gGT, total protein, glucose, NEFA, Mg, Ca, P, and serotonin (Table S1).

**Table 1.** Portal vein plasma biomarker concentration (only when *P* < 0.15 for at least one main effect or the interaction) at 1-wk after weaning for weanling pigs (*n* = 24) with high or low FI for d1 till d3 (FId1-3) which was followed by either a high or low FI for d4 till d6 (FId4-6) after weaning resulting in 4 FI classes HH, HL, LH, and LL

	FI classes				Pooled SE^1^	*P*-values		
	HH	HL	LH	LL		FId1-3	FId4-6	FId1-3 × FId4-6
Haptoglobin, g/L	0.47	1.17	0.78	1.17	0.153	0.165	0.006	0.207
PigMAP, g/L	0.66	0.89	0.84	1.21	0.102	0.052	0.021	0.826
Cortisol, ng/mL	18.3	15.9	22.2	20.8	3.29	0.100	0.639	0.772
IGF1, ng/mL	161	122	90.2	85.7	9.35	<0.001	0.108	0.256
Triglycerides, mg/dL	26.2	19.0	28.6	27.0	3.63	0.081	0.311	0.307
Albumin, g/L	28.1	24.0	27.1	27.0	0.97	0.331	0.093	0.105
Creatinine, umol/L	91.7^xy^	81.8^y^	94.3^xy^	103^x^	3.79	0.015	0.767	0.052
Urea, mmol/L	0.67	0.60	0.73	0.82	0.058	0.050	0.991	0.285
Bilirubin, umol/L	3.07	3.45	2.58	3.55	0.237	0.427	0.022	0.269
Bile acids, µmol/L	6.61	18.3	5.36	18.69	5.265	0.860	0.049	0.833
ALP, IU/L	243	188	193	172	17.20	0.113	0.100	0.518
GLDH, IU/L	2.97^ab^	9.90^a^	6.38^ab^	1.95^b^	2.319	0.325	0.987	0.029
LDH, IU/L	1,100	1,307	1,270	1,588	133.7	0.044	0.101	0.776

^1^Pooled standard error of the mean for the interaction.

^a,b^LSMEANS of the interaction without a common superscript differ at *P* < 0.05.

^x,y^LSMEANS of the interaction without a common superscript tend to differ at *P* < 0.10.

### Organ weights, intestinal morphometrics, biogenic amines, and ALPi

There were no significant interactions between FI d1 to d3 and d4 to d6 for the organ weights or jejunal histomorphometry traits (Table 2). However, SI, pancreas, and colon plus rectum increased (all *P* < 0.04) for the high FI d1 to d3 compared to low FI d1 to d3 pigs. Similarly, villus length increased (*P* = 0.002), and crypt depth (*P* = 0.076) and surface area (*P* = 0.051) tended to increase for the high FI d1 to d3 compared to low FI d1 to d3 pigs. Regarding FI d4 to d6, a high FI increased villus length (*P* = 0.033) and villus length to crypt depth ratio (*P* = 0.046) compared to a low FI. Alkaline phosphatase in jejunal tissue showed an interaction (*P* = 0.018) between FI classes where HL pigs had higher ALPi concentration than HH and LL with LH being intermediate and not different (Table 2). However, there were no significant differences in ALPi concentration in ileal tissue (*P* = 0.946). Regressions between plasma ALP and ALPi showed a weak but significant association in ileum (R^2^ = 0.20; *P* < 0.01), whereas there was no association with the ALPi concentration in jejunum (*P* > 0.05).

**Table 2.** Organ weights, jejunal histomorphometry, alkaline phosphatase (ALPi) in jejunal and ileal tissue, and biogenic amines and CP in complete SI content at 1-wk after weaning for pigs with high or low FI for d1 till d3 (FId1-3) which was followed by either a high or low FI for d4 till d6 (FId4-6) after weaning resulting in four FI classes HH, HL, LH, and LL

	FI classes				Pooled SE^1^	*P*-values		
	HH	HL	LH	LL		FId1-3	FId4-6	FId1-3 × FId4-6
Stomach, g/kg BW	3.95	3.76	4.11	3.81	0.12	0.496	0.099	0.707
Small intestine, g/kg BW	35.8	35.2	28.8	28.8	1.69	0.003	0.884	0.853
Pancreas, g/kg BW	1.79	1.76	1.58	1.53	0.08	0.033	0.697	0.928
Colon and rectum, g/kg BW	14.8	15.1	13.7	12.1	0.82	0.029	0.574	0.333
*Jejunum*								
Villus length, μm	451	389	363	327	21.4	0.002	0.033	0.555
Crypt depth, μm	204	220	194	185	11.8	0.076	0.768	0.306
Villus length: crypt depth	2.31	1.84	1.94	1.83	0.131	0.159	0.046	0.196
Surface area villi, mm^2^	39.3	42.0	34.5	31.8	3.62	0.051	0.995	0.468
ALPi, IU/g tissue	21.8^b^	31.8^a^	25.4^ab^	21.8^b^	2.54	0.194	0.366	0.018
*Ileum*								
ALPi, IU/g tissue	18.9	15.1	19.2	15.1	2.55	0.975	0.120	0.946
*Small intestine contents*								
Total wet content, g/kg BW	17.0	22.3	17.3	17.3	2.09	0.347	0.292	0.289
Cadaverine, µg/kg	473	679	795	1,216	107.3	<0.001	0.005	0.802
Histamine, µg/kg	6,960	5,578	23,490	33,010	8,252	0.002	0.921	0.502
Putrescine, µg/kg	1,751	1,823	2,613	2,226	299	0.088	0.723	0.556
Spermidine, µg/kg	4,405	5,220	5,789	5,711	848	0.321	0.702	0.638
Spermine, µg/kg	748	1,108	703	977	218	0.679	0.266	0.899
Crude protein, g/100 g DM	14.9	14.7	15.0	14.4	0.65	0.851	0.579	0.757

^1^Pooled standard error of the mean for interaction.

^a,b^LSMEANS of the interaction without a common superscript differ at *P* < 0.05.

Looking at the biogenic amines there were no significant interactions between FI d1 to d3 and d4 to d6 and only a few main effects were found (Table 2). Cadaverine and histamine increased (*P* < 0.005) and putrescine tended to increase (*P* = 0.088) for low FI d1 to d3 compared to high FI d1 to 3 while cadaverine also increased (*P* = 0.005) for low FI d4 to d6 compared to high FI d4 to 6.

### Jejunal gene expression

Table 3 reports the main differences in jejunal transcription of several genes related to various intestinal functions between the FI d1 to d3 and FI d4 to d6 classes and their interaction. For readability, among the 44 target genes, only the 24 showing a *P* < 0.15 for any of the two main effects or the interaction were reported. *SOD2m* gene showed tendencies for increased jejunal expression in pigs with low FI relative to high FI for both main effects FI d1 to d3 (*P* = 0.059) and FI d4 to d6 (*P* = 0.063) without an interaction between the two periods (*P* = 0.320). *MUC13* had a higher expression for LH compared to HH (*P* = 0.027) with HL and LL being intermediate and not different. The *AKT1S1* gene, was downregulated in HL compared to HH and LL (*P* = 0.007) with LH being intermediate and not different. The *IGF1R* gene transcription was downregulated in HL relative to LL, with LH not different, and HH being expressed the highest among all (*P* = 0.001). The *SLC39A4_ZIP4*, important in cellular zinc homeostasis and zinc transport, was upregulated for HL relative to HH, LH, and LL (*P* = 0.018). The transcription of *HNMT* gene, which inactivates histamine, was upregulated for high FI d1 to d3 compared to low FI d1 to d3 (*P* = 0.037). The *SLC13A1_NAS1* gene abundance, related to sulfate reabsorption, was marginally (*P* = 0.051) increased for low FI d4 to d6 compared to high FI d4 to d6.

**Table 3.** Gene expression^1^ (only when *P* < 0.15 for at least one main effect or the interaction) in jejunal tissue at 1-wk after weaning between pigs with high or low FI for d1 till d3 (FId1-3) which was followed by either a high or low FI for d4 till d6 (FId4-6) after weaning resulting in four FI classes HH, HL, LH, and LL

		FI classes				Pooled SE^1^	*P*-values		
Gene function		HH	HL	LH	LL		FId1-3	FId4-6	FId1-3 × FId4-6
Antioxidant	SOD2m	0.96	1.03	1.01	1.22	0.063	0.059	0.063	0.320
Barrier	MUC13	0.72^b^	1.02^ab^	1.10^a^	0.97^ab^	0.091	0.063	0.272	0.027
	OCLN	0.94	1.14	1.11	1.11	0.068	0.266	0.137	0.139
Digestive, endocrine, and metabolic	AKT1S1	1.23^a^	0.99^b^	1.05^ab^	1.19^a^	0.064	0.804	0.530	0.007
	ALPi	0.96^y^	1.34^x^	1.32^x^	1.04^xy^	0.174	0.826	0.746	0.069
	ANPEP	0.88^y^	1.15^x^	1.01^y^	0.90^y^	0.100	0.613	0.458	0.082
	HNMT	0.83^y^	0.98^x^	0.83^y^	0.79^y^	0.061	0.037	0.467	0.073
	IGF1R	1.24^a^	0.88^c^	1.02^cb^	1.15^b^	0.076	0.388	0.114	0.001
	SLC13A1_NAS1	0.64	0.95	0.74	2.42	0.448	0.169	0.051	0.300
	SLC15A1_PEPT1	0.98^y^	1.34^x^	1.18^xy^	1.09^xy^	0.134	0.863	0.337	0.056
	SLC39A4_ZIP4	0.88^b^	1.44^a^	0.92^b^	0.90^b^	0.104	0.048	0.028	0.018
	SLC16A1_MCT1	1.00^x^	0.85^y^	0.86^y^	0.93^xy^	0.058	0.704	0.482	0.070
Immune and inflammatory	FAXDC2	0.28^b^	1.14^a^	0.85^a^	0.85^a^	0.257	0.151	0.064	0.039
	IL1β	1.39	1.33	0.92	1.15	0.231	0.071	0.668	0.431
	IL-10	1.29	0.94	0.85	0.76	0.183	0.108	0.265	0.594
	IL-6	0.75	0.71	0.62	0.40	0.098	0.005	0.147	0.141
	MyD88	1.03^b^	1.25^a^	1.10^ab^	1.07^ab^	0.051	0.317	0.115	0.027
	S100A9	1.56	0.60	0.88	0.67	0.253	0.288	0.051	0.171
	TGF-β1	1.19	1.08	0.84	0.84	0.075	0.001	0.533	0.564
	TLR2	0.93	0.65	0.44	0.50	0.096	0.004	0.486	0.120
	TLR4	1.46^x^	1.18^xy^	0.92^y^	0.99^y^	0.085	0.001	0.323	0.078
	TNF-α	0.99	1.19	0.84	0.89	0.137	0.058	0.454	0.606
Stress response	CRHR1	1.29^x^	0.74^y^	0.68^y^	1.30^x^	0.357	0.891	0.921	0.078
	HSD11B1	1.36^a^	0.97^ab^	0.74^b^	0.97^ab^	0.131	0.030	0.797	0.030

^1^Gene expression values are presented as ratios of cycle relative threshold value for each gene normalized to that of the reference sample and analyzed using log transformation.

^2^Pooled standard error of the mean for the interaction.

^a,b^LSMEANS without a common superscript differ at *P* < 0.05.

^x,y^LSMEANS without a common superscript tend to differ at *P* < 0.10.

The immune system antigen recognition and inflammatory function showed a general gene upregulation in pigs with a high initial FI (HL + HH) relative to their low counterparts (LH + LL) as indicated by an increased transcription for *IL-6* (*P* = 0.005), *TGF-β1* (*P* = 0.001), *TLR2* (*P* = 0.004), *TLR4* (*P* = 0.001), *IL1β* (*P* = 0.071), and *TNF-α* genes (*P* = 0.058). The abundance of *S100A9* gene, which modulates the inflammatory response and leukocyte recruitment, tended to be (*P* = 0.051) increased in high FI d4 to d6 pigs compared to their low FI counterparts. In addition, an interaction between FI d1 to d3 and FI d4 to d6 FI was found for *FAXDC2* being downregulated in HH pigs relative to HL, LH, and LL pigs (*P* = 0.039). The *MyD88* gene, a universal adapter to activate the transcription of nuclear factor kappa B subunit 1 (NF-κB) through toll-like receptors, was upregulated for HL pigs relative to HH pigs (*P* = 0.027), with LH and LL being intermediate and not different. Finally, the *HSD11B1* gene, which catalyzes the conversion of cortisone to cortisol and is involved in regulating the stress response, had a higher jejunal expression for HH pigs than LH pigs (*P* = 0.030) with HL and LL pigs being intermediate and not different.

### Integrative ADFI, immune biomarkers, and gastrointestinal tract development

**Figure 2.**
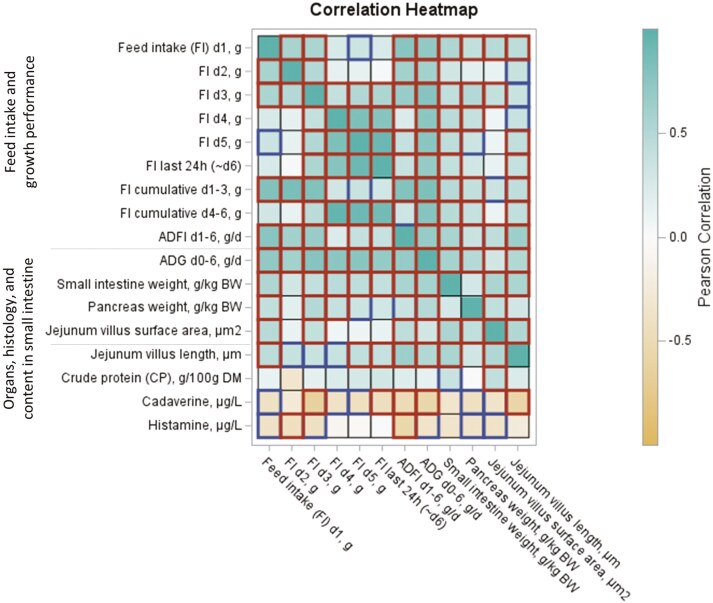
Heatmap for Pearson r correlation (*P*-values < 0.05 are indicated by the red boxes whereas *P*-values < 0.10 are in blue boxes) between the daily FI of individual piglets (*n* = 24) and the variables correlating at *P* < 0.10 with FI including: growth performance traits, gastrointestinal organ weights, histomorphometry traits, and amino acid and CP fermentation products in SI content.

As shown in the correlation Figure 2, except for d3, daily FI measurements positively correlated (*P* < 0.05) within d1 to d3 and d4 to d6 but not between these two periods. Only by d3, pigs had a clear and consistent FI pattern as FI on d3 correlated positively with the FI of all other days FI (*P* < 0.05). When looking at daily correlations between pigs’ FI and gastrointestinal development, the FI level for all days except for d2 (*P* > 0.05) was highly correlated to both the SI and pancreas weights (*P* < 0.05). Similarly, high FI on d1, d3, d5, and d6 was correlated to increased jejunum villus surface area and villus length (*P* < 0.05). A low FI on d2 was correlated to a higher histamine concentration in SI digesta at 1 wk after weaning (*P* < 0.05). The lower the FI on d3 and the lower the FI the last 24h before sampling (~d6), the higher the cadaverine concentration in SI content (*P* < 0.05). Another correlation heatmap was created to depict correlations between daily FI, the immune and metabolic biomarkers in plasma, jejunal gene expression, and main intestinal development traits (Fig. S3). Data highlighted the importance of FI onset by the day and served to validate the chosen biomarkers. For instance, pigs with high FI by d1 already correlated positively with IGF1 and negatively with plasma creatinine on d6 (*P* < 0.05; Fig. S3).

**Figure 3.**
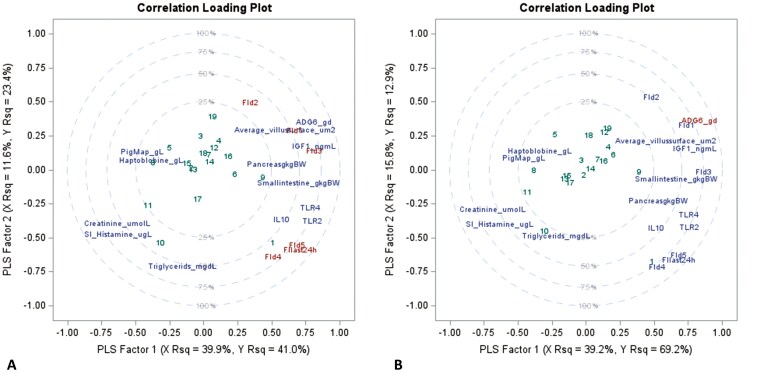
Correlation loading plot for partial least squares regression (PLS) with two extracted factors for the most important variables (The variables included in the final PLS were selected based on preliminary screening using significant Pearson correlations. Once the first models were created, further selection was done keeping the most important variables based on the importance plot from PROC PLS in SAS) explaining day-to-day FI (A) and ADG (B) the first week after weaning.

Finally, all data were combined to identify the key biomarkers using PLS. The day-to-day FI (Fig. 3A) clustered close together in the first explanatory factor (x axis, 41% R^2^) while the second factor (y axis, 23.4% R^2^) included dispersion and grouped separately d1 till d3 and d4 till d6, matching previous observations. The most relevant positive variables to explain day-to-day FI were villus surface area in jejunum, ADG d0 to d6, and IGF1 in portal plasma and the most relevant negative ones were creatinine in portal plasma and histamine in SI content followed by PigMAP, haptoglobin, and triglycerides in portal plasma. The PLS for ADG d0 to d6 (Fig. 3B) explained 82% of the variance (factor 1 = 69.2% R^2^ and factor 2 = 12.9% R^2^). The daily FI, with d1 having the highest importance among days, together with villus surface area, SI weight, and IGF1 in portal plasma correlated positively to ADG. In addition, histamine in SI content and creatinine, PigMAP, triglycerides, and haptoglobin in portal plasma showed strong negative correlations to ADG d0 to d6.

## Discussion

The present study was aimed at characterizing associations between daily FI during the first days after weaning and the effects on key biomarkers of intestinal physiology, systemic metabolism, and immunity of pigs at 1-wk postweaning.

Our previous studies (Fabà et al., 2024a, 2024b) have shown that variations in the early FI of pigs affected growth rate during the entire nursery while impacting gut health. In the present study, we classified piglets based on their FI d1 to d3 and d4 to d6 yielding HH, HL, LH, and LL levels of FI. Based on this classification, the weight gain was only higher for the HH and HL pig (i.e., high FI d1 to d3) compared to LH and LL (i.e, low FI d1 to d3), which concurs with early FI patterns impacting weight gain (Engelsman et al., 2023; Fabà et al., 2024a). However, the unexpected lack of weight gain differences between HH and HL, as well as between LH and LL, may be explained by increased water retention (edema) rather than tissue accretion. As such, variability in weaning-associated edema relates to hypophosphatemia (Van Kempen et al., 2023). We can speculate a general hypophosphatemia as 55% and 71% of all pigs had P (median = 2.57 mmol/L) and ALP (median = 200 IU/L) plasma levels below the 95% confidence interval previously described (2.6 to 3.8 mmol/L and 233 to 1332 IU/L, respectively; Perri et al., 2017). Plasma P levels were not significantly different between feeding patterns, but HL pigs had 10% lower levels than HH ones, and LL pigs had 7% lower levels than LH ones. The small sample size may explain the lack of significance. Additionally, low P can cause weight gain via edema while adequate P supports tissue accretion (Van Kempen et al., 2023), adding confounding. In effect, plasma IGF1, being higher in high FI d1 to d3 pigs, correlated positively with P, organ weights, and jejunal villus metrics, indicating the need for P for metabolic activity and growth. Altogether, increasing FI after the first few days following weaning (d4 to d6) may help overcome postweaning edema while d1 to d3 FI was more important for growth.

Pigs primarily rely on glycolysis, gluconeogenesis, and lipolysis pathways to create metabolic energy early after weaning (Han et al., 2016; Southey et al., 2021). Our results show evidence of this activation of secondary energy pathways, with increased plasma creatinine levels for low FI d1 to d3 pigs. This pathway occurs mainly in the liver and at the expense of amino acids, such as arginine, glycine, and methionine (Wyss and Kaddurah-Daouk, 2000; Brosnan et al., 2011). The elevated plasma LDH observed in our study is a rather nonspecific finding, but muscle and liver damage may cause an increase in plasma LDH (Keller et al., 1974). The anaerobic metabolism and liver disorders in the context of weaning (Jayaraman and Nyachoti, 2017; Klein et al. 2020; Southey et al., 2021), plus the extreme contrast in FI here, may explain the LDH increase. Altogether, pigs with low FI early after weaning had a fasting-like metabolism and hepatic dysfunction while they strongly rely on amino acids for energy and glucose production.

One may anticipate LL pigs to struggle the most in the immediate postweaning phase, but our data on biomarker responses for metabolic stress shows that they are not necessarily the only ones. The consequences of an erratic feeding pattern were captured by the GLDH mitochondrial enzyme which was above reference (0-9 IU/L, 1-2 d preweaning, Perri et al., 2017) for 17% of the pigs and mainly associated with HL being higher than LL. Circulating GLDH rise during oxidative stress (Plaitakis et al., 2017) indicating a possible cause for transitioning from high to low FI. In addition, GLDH is essential for the energy supply of the liver, kidney, brain, and pancreatic islets when glucose is limited due to the catabolic process of converting glutamate to α-ketoglutarate which enters the Krebs cycle (Stanley, 2009; Plaitakis et al., 2017). Instead of entering a recovery phase, pigs with low d4 to d6 FI continued to struggle and showed a systemic acute inflammatory response [increased haptoglobin and PigMAP corresponding to previous studies (Petersen et al., 2004; Heegaard et al., 2011)]. Pigs with low FI d4 to d6 also exhibited decreased plasma albumin levels, indicating compromised growth, general health, nutritional status, and liver function (Doornenbal et al., 1986; Wang et al., 2020; Rymut et al., 2021). Furthermore, the observed higher plasma bilirubin levels in these pigs may be linked to a disrupted liver-gut axis signaling (Lu et al., 2014; Hamoud et al., 2018), while the increased plasma bile acids could relate to intestinal permeability and inflammation (Calzadilla et al., 2022). None of the pigs showed any pathological findings in their macroscopic appearance during dissection, suggesting that the results were not related to disease. Hence, regardless of FI d1 to d3, pigs can continue with an erratic FI from d4 to d6 and metabolic stress.

In general, HH pigs showed an improved intestinal function demonstrating that a high and rapid increase in daily FI can be beneficial. A recognizable example was *IGF1R*, which was upregulated in HH pigs, with enhanced signaling from circulating *IGF1* observed to be improving intestinal tissue growth and even protecting it from necrosis (Bohin et al., 2020; Yan et al., 2022). *FAXDC2*, a membrane component involved in lipid metabolism and cell differentiation (Madan et al., 2024), may indicate a compensatory response for intestinal epithelium recovery as it was found to be upregulated in all groups except HH. Moreover, HH pigs had upregulated *HSD11B1*, which could be a local anti-inflammatory regulation via the re-activation of cortisone to cortisol and glucocorticoid signaling as described in murine models of polyarthritis (Fenton et al., 2021). At the other extreme, LL pigs were expected to show a pro-inflammatory and metabolically disrupted profile in jejunal gene expression. However, and similarly to GLDH results, HL pigs but not LL pigs showed downregulation for various genes with negative implications. For instance, genes *IGF1R* and *AKT1S1*, the latter coding a subunit protein of *mTORC1* (Wang et al., 2008), were downregulated for HL compared to LL. As discussed earlier, a lower *IGF1* signaling may be associated with hampered intestinal growth and cell proliferation. Regarding *AKT1S1*, in absence of insulin and nutrients, this gene associates with and inhibits *mTORC1* activity by blocking the receptor substrate-recruitment site (Yang et al., 2017). Alterations to *mTORC1* processes in HL may hinder protein synthesis and cell growth but also impair nutrient sensing and utilization in the jejunum (Wang et al., 2008; Yang et al., 2017). The HL pigs also appear to make compensatory efforts such as 1) increased *ALPi* as LPS detoxifying enzyme on the disrupted villi’s brush border (Lackeyram et al., 2010), 2) *HNMT* upregulation as inactivating response to histamine (Smolinska et al., 2022), and 3) increased gene expression of *SLC39A4_ZIP4*, which may be aiming to enhance Zn absorption since Zn is essential for several cellular processes and immune function (Virden et al., 2002; Ou et al., 2007; Suttle, 2010). Altogether, the high-to-low erratic feeding pattern was the one with the most disturbed and upregulated intestinal metabolic function.

A negative circular response was hypothesized where low initial FI leads to metabolic dysfunction, poor intestinal health, and local inflammation which, in turn, further reduces FI. However, in contrast, initiate signaling pathways to mount an effective immune response (*TRL2* and *TLR4*), pro-inflammatory (*IL1β*), and anti-inflammatory and modulatory genes (*IL-10, TGF-β1, and IL-6*) were increased in jejunal tissue for pigs with a high FI during d1 to d3. In a similar paradox, Engelsman et al. (2023) reported that pigs with high early FI increased systemic inflammation and alongside showed improved growth at the end of the nursery. To some extent, this challenges the paradigm that low FI during the first days after weaning leads to increased local inflammation (Pié et al. 2004; Lallès et al., 2007). Multiple correlations and the PLS analyses revealed that an activated immune system in jejunum was correlated with improved metrics including weight gain, SI weight, jejunal morphometry, and plasma *IGF1*. Using 35-d-old pigs that had already passed the weaning stress, Wang et al. (2025) recently demonstrated that 7-d severe feed restriction upregulated jejunal pro-inflammatory cytokines *IL-17* and *IL-22*. However, the effect was absent when feed restriction occurred for 3 d. It could be that a thin line separates the low voluntary FI pigs with initial and subtle local inflammatory response from a vigorous local immune reaction associated with the weaning event. For instance, the *MyD88* gene was upregulated in HL compared to HH while their jejunal immune profiles were similar. This gene is involved in *TLR* and *IL1* receptor signaling in the innate immune response and *NFK-β* activation, cytokine secretion, and the inflammatory response (Liu et al., 2023). Consequently, some pathways in the pro-inflammatory response may be associated with reducing FI, while the general inflammation-like profile observed here may concur with weaning and a high exposure to antigens associated with high FI endorsing intestinal maturation.

Early research already attributed an intestinal inflammatory-like profile to the dietary antigens combined with weaning-associated damage on intestinal mucosa (McCracken et al., 1999). Our findings complement the intestinal maturation and cytokine gene responses described by Pié et al. (2004), with an early and a transient period. However, rather than a general time-trend, intestinal maturation and mRNA responses could be differentiated by the initial postweaning feeding patterns. For instance, an upregulation in *IL-1β* as response to lipopolysaccharide from gram-negative bacteria signaling the *TLR4* could be stronger, as more effective and efficient in high initial FI pigs. Even so, early upregulation of *IL-1β* could mediate tissue repair and resolution of inflammation (Bersudsky et al., 2014). Wang et al. (2025) reported that various genes, including the barrier function *MUC2*, were downregulated by a 3-d feed restriction, indicating no short-term effect of fasting on inflammation. Similarly, in a 5-d fasting geese study, immune genes such as *IgA* and *IFN-γ* were downregulated, while *IgG, TNF-α, IL-6*, and *IL-10* remained stable (Liu et al., 2025). The precise impacts of nutrient availability remain unclear, and our day 6 measurements lack longitudinal data to distinguish time-driven from FI-driven maturation processes. Recent research describes a complex and beneficial effect in intestinal maturation and the vigorous immune reaction to dietary antigens, microbiota, and metabolites (Nabhani et al., 2019). Both hypotheses, vigorous immune reaction and inflammation, can coexist while the presence of unabsorbed protein and the risk of dysbiosis in weaned pigs may determine the gut health outcome (Kim et al., 2012; Zhang et al., 2020; Li et al., 2022). This is partly supported by our histamine and cadaverine data presented here. Altogether, high early FI and the intestinal maturation with weaning-related immune reactions may obscure local inflammatory pathways impacting behavior and FI, especially in HL and LL pigs, that correlate to the systemic acute inflammation and reduced performance seen in these pigs.

## Conclusion

An early, high, and gradual increase in FI during the first week after weaning yields a more robust metabolic and intestinal physiology profile explaining an improved growth. In addition, a high initial (d1 to d3) FI was associated with an upregulated intestinal immune response possibly due to higher nutrient availability, effective mRNA response, and vigorous immune reaction to dietary antigens, microbiota, and metabolites. Regarding our hypothesis, pigs transitioning from high initial FI to a low pattern showed distinct metabolic alterations, but their subtle local inflammation was masked by the vigorous immune response in all pigs with high initial FI. Furthermore, a sustained struggle with low FI during d4 to d6 elicited a systemic inflammatory response and further compromised markers of intestinal and systemic metabolic functions. Results from the present study are relevant for future research aiming to improve general health and robustness of newly weaned pigs including a list of relevant biomarkers that are associated with individual FI.

## Supplementary Material

skaf099_suppl_Supplementary_Materials

## Abbreviations:

ADFI: average daily feed intake
ADG: average daily gain; AKT1S1, proline-rich AKT1 substrate 1
ALP: alkaline phosphatase
ALPi: intestinal alkaline phosphatase
ALT: alanine aminotransferase
AST: aspartate aminotransferase
BW: body weight
CCK: cholecystokinin
CP: crude protein
CPK: creatine phosphokinase;
CRHR1: corticotropin releasing hormone receptor
DM: dry matter
EFS: electronic feeding station
FAXDC2: fatty acid hydroxylase domain containing 2
FI: feed intake
gGT: gamma glutamyl transpeptidase
GLDH: glutamate dehydrogenase
HNMT: histamine N-methyltransferase
HSD11β1: hydroxysteroid (11-beta) dehydrogenase
IGF-I: insulin-like growth factor
IGF1R: IGF-I receptor
IL: interleukin
LDH: lactate dehydrogenase
MUC: mucins
MyD88: Myeloid Differentiation Primary Response Protein
NE: net energy
NEFA: non-esterified fatty acids
NFK-β: nuclear factor kappa-beta
OCLN: occludin
PigMAP: Pig Major Acute-phase Protein
PLS: partial least squares regression
SI: small intestine
SOD2: Superoxide dismutase
TGF-β1: transforming growth factor beta 1
TLR: Toll-like receptor
TNFα: tumor necrosis factor α
ZO1: zonula occludens 1
