# Supplementary material for: Impact of initial postweaning feed intake on weanling piglet metabolism, gut health, and immunity
Source: J Anim Sci. 2025 Mar 31;103:skaf099. doi: 10.1093/jas/skaf099 (PMC12082826; doi:10.1093/jas/skaf099)
Supplement: skaf099_suppl_Supplementary_Materials [file skaf099_suppl_supplementary_materials.zip › Fabà et al 2024 Early feed intake follow up - Suplementary material 10JULY2024 SMO.docx]

**Impact of Initial Feed Intake on Piglet Metabolism, Gut Health, and Immunity**

Lluís Fabà*†, Susana M. Martín-Orúe±, Tetske G. Hulshof†, José Francisco Pérez±, Michael O. Wellington†, and Hubèrt M. J. Van Hees†‡

†Trouw Nutrition R&D, Swine Research Centre, Veerstraat 38, 5831 JN, Boxmeer, The Netherlands

‡Ghent University, Faculty of Veterinary Medicine, Department of Veterinary and Biosciences, Merelbeke, Belgium

±Animal Nutrition and Welfare Service, Department of Animal and Food Sciences, Universitat Autònoma de Barcelona, 08193 Bellaterra, Spain

**Table S1**. Additional (non-responsive) portal vein plasma biomarker concentration at 1-week after weaning for weanling pigs (n = 24) with or low feed intake for d1 till d3 (FId1-3) which was followed by either a high or low FI for d4 till d6 (FId4-6) after weaning resulting in four FI classes HH, HL, LH, and LL.

|  | **Feed intake classes** | | | | ***Pooled***  ***SE^1^*** | **P-values** | | |
| --- | --- | --- | --- | --- | --- | --- | --- | --- |
|  | **HH** | **HL** | **LH** | **LL** |  | **FI d1-3** | **FId4-6** | **FId1-3 × FId4-6** |
| ALT, IU/L | 33.7 | 29.8 | 28.0 | 32.0 | *2.608* | 0.571 | 0.950 | 0.214 |
| AST, IU/L | 52.5 | 76.4 | 55.2 | 81.9 | *14.08* | 0.699 | 0.125 | 0.958 |
| CPK, IU/L | 531 | 337 | 411 | 412 | *73.2* | 0.890 | 0.281 | 0.276 |
| gGT, IU/L | 32.3 | 30.8 | 35.5 | 31.0 | *3.30* | 0.581 | 0.477 | 0.665 |
| Total protein, g/L | 45.0 | 44.3 | 46.3 | 45.4 | *1.18* | 0.300 | 0.574 | 0.938 |
| Glucose, mg/dL | 183 | 163 | 171 | 211 | *14.3* | 0.320 | 0.635 | 0.108 |
| NEFA, mmol/L | 0.10 | 0.10 | 0.10 | 0.11 | *0.008* | 0.576 | 0.446 | 0.839 |
| Magnesium, mmol/L | 0.84 | 0.87 | 0.86 | 0.86 | *0.052* | 0.890 | 0.871 | 0.745 |
| Calcium, mmol/L | 2.66 | 2.60 | 2.71 | 2.54 | *0.062* | 0.892 | 0.105 | 0.315 |
| Phosphorus, mmol/L | 2.78 | 2.52 | 2.60 | 2.44 | *0.142* | 0.430 | 0.264 | 0.801 |
| Serotonin ng/mL | 190 | 247 | 220 | 82 | *70.3* | 0.308 | 0.436 | 0.187 |

^1^Pooled standard error of the mean for interaction.

^2^ Abbreviations: ALT = pig major acute-phase protein; AST = insulin-like growth factor 1; CPK = creatine phosphokinase; gGT = Alkaline phosphatase; NEFA = phosphate.


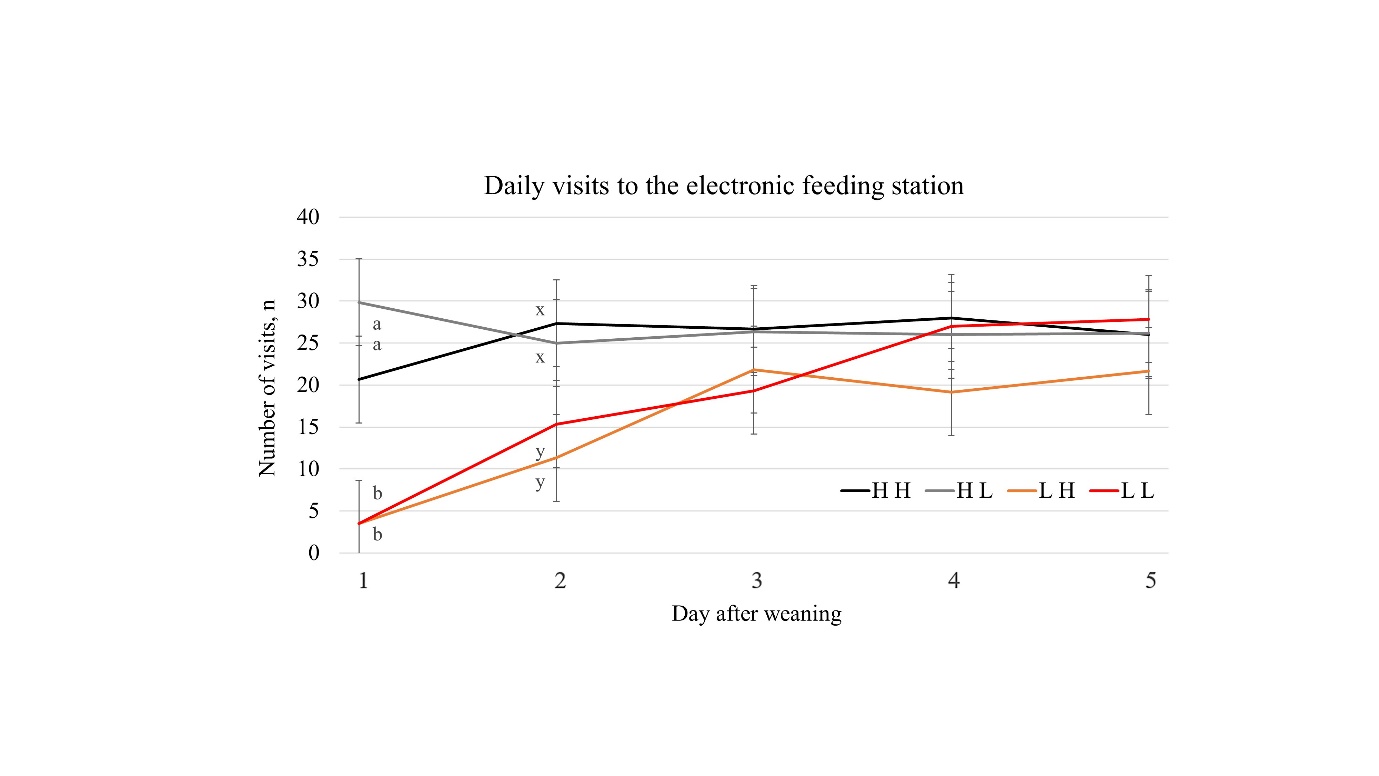


**Figure S1.** Daily number of visits to the electronic feeding station during d1 till d5 after weaning of pigs with high or low feed intake for d1 till d3 which was followed by either a high or low FI for d4 till d6 after weaning (HH, HL, LH and LL, respectively).

^a-b^LSMEANS within a day without a common superscript differ at P < 0.05.

^x-y^LSMEANS within a day without a common superscript tend to differ at P < 0.10.

The daily visits to the feeder between the four classes were higher on d1 (P < 0.01) and tended to be higher on d2 (P < 0.10) for HH and HL compared to LH and LL (Figure S1).


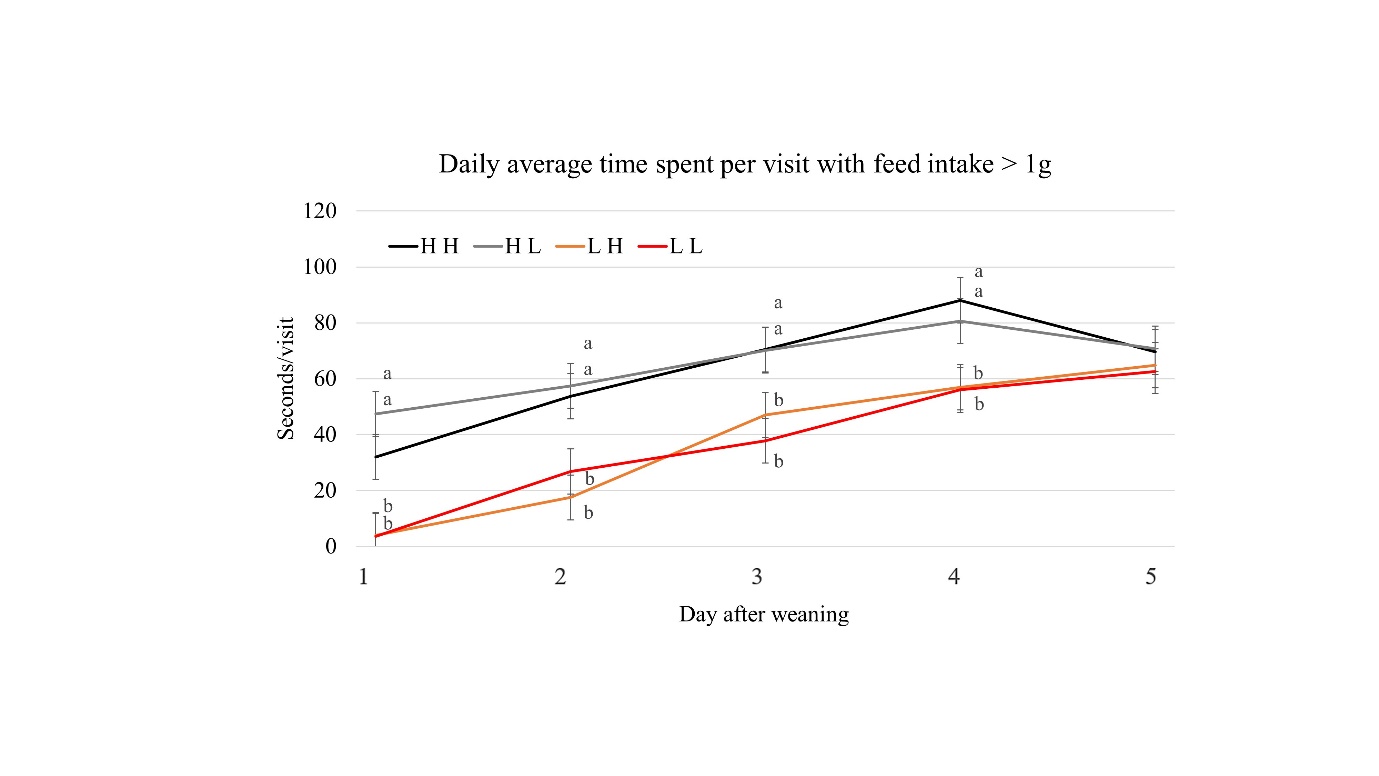


**Figure S2.** Daily average time spent per visit to the electronic feeding station during d1 till d5 after weaning of pigs with high or low feed intake for d1 till d3 which was followed by either a high or low FI for d4 till d6 after weaning (HH, HL, LH and LL, respectively).

^a-b^LSMEANS within a day without a common superscript differ at P < 0.05.


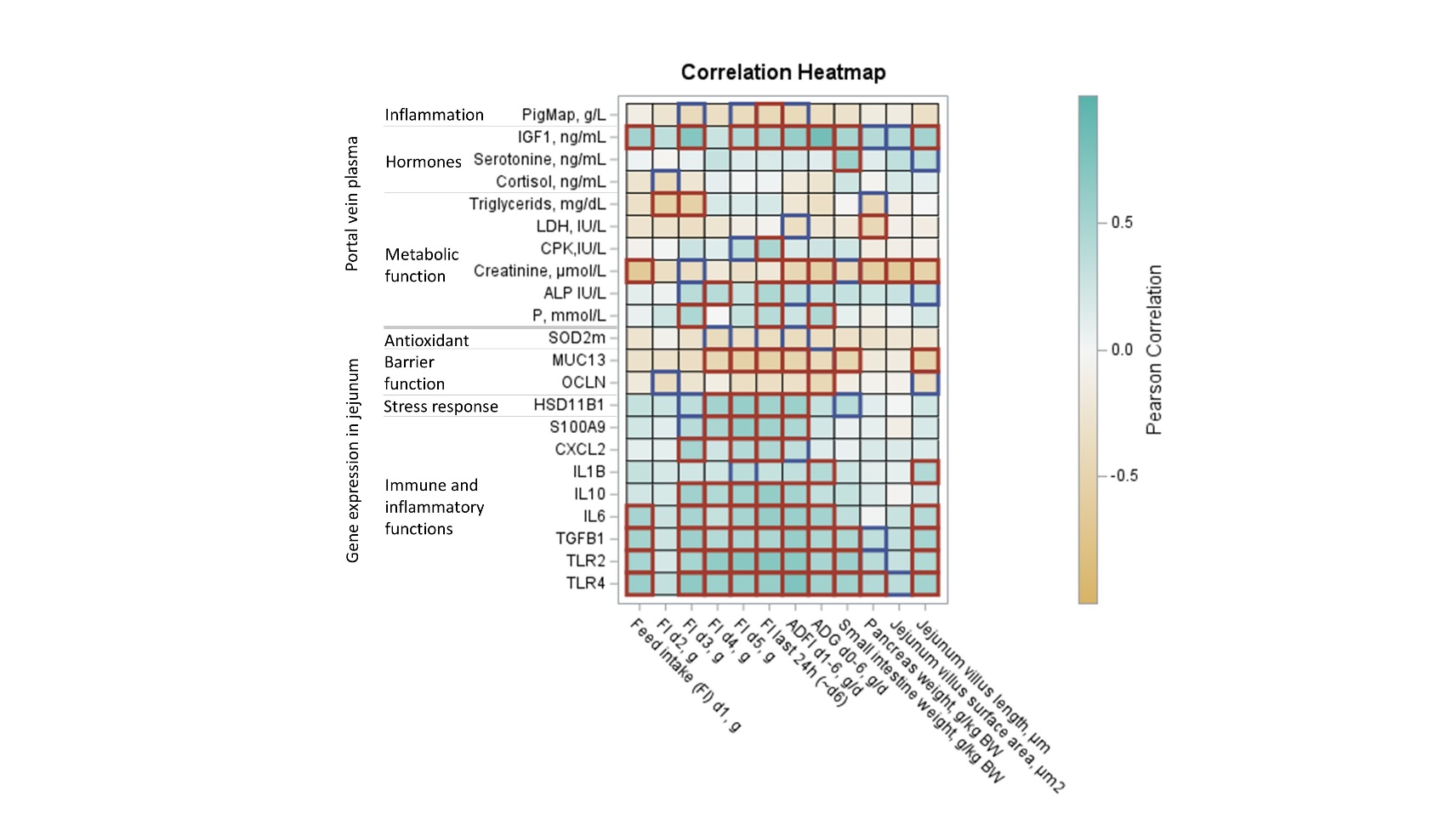


**Figure S3.** Heatmap for Pearson r correlation^1^ between the daily feed intake (FI) of individual pigs (n = 24) and the variables correlating at P < 0.10 with FI including: growth performance traits, gastrointestinal organ weights, histomorphometry traits, portal vein plasma biomarkers, or jejunal gene expression for various gastrointestinal functions^2^.

^1^P values < 0.05 are indicated by the red boxes whereas P values < 0.10 are in blue boxes.

^2^ Abbreviations: PigMap = pig major acute-phase protein; IGF1 = insulin-like growth factor 1; LDH = lactate dehydrogenase; CPK = creatine phosphokinase; ALP = Alkaline phosphatase; P = phosphate; SOD2m = superoxide dismutase 2 mitochondrial; MUC13 = mucins 13; OCLN = occludin; HSD11B1 = hydroxysteroid 11-beta dehydrogenase 1; S100A9 = S100 calcium binding protein A9; CXCL2 = C-X-C motif chemokine ligand 2; IL1B = interleukin 1 beta; IL10 = interleukin 10; IL6 = interleukin 6; TGFB1 = transforming growth factor beta 1; TLR2 = toll like receptor 2; and TLR4 = toll like receptor 4.
