# Supplementary figures and images for: Impact of initial postweaning feed intake on weanling piglet metabolism, gut health, and immunity
Source: J Anim Sci. 2025 Mar 31;103:skaf099. doi: 10.1093/jas/skaf099 (PMC12082826; doi:10.1093/jas/skaf099)

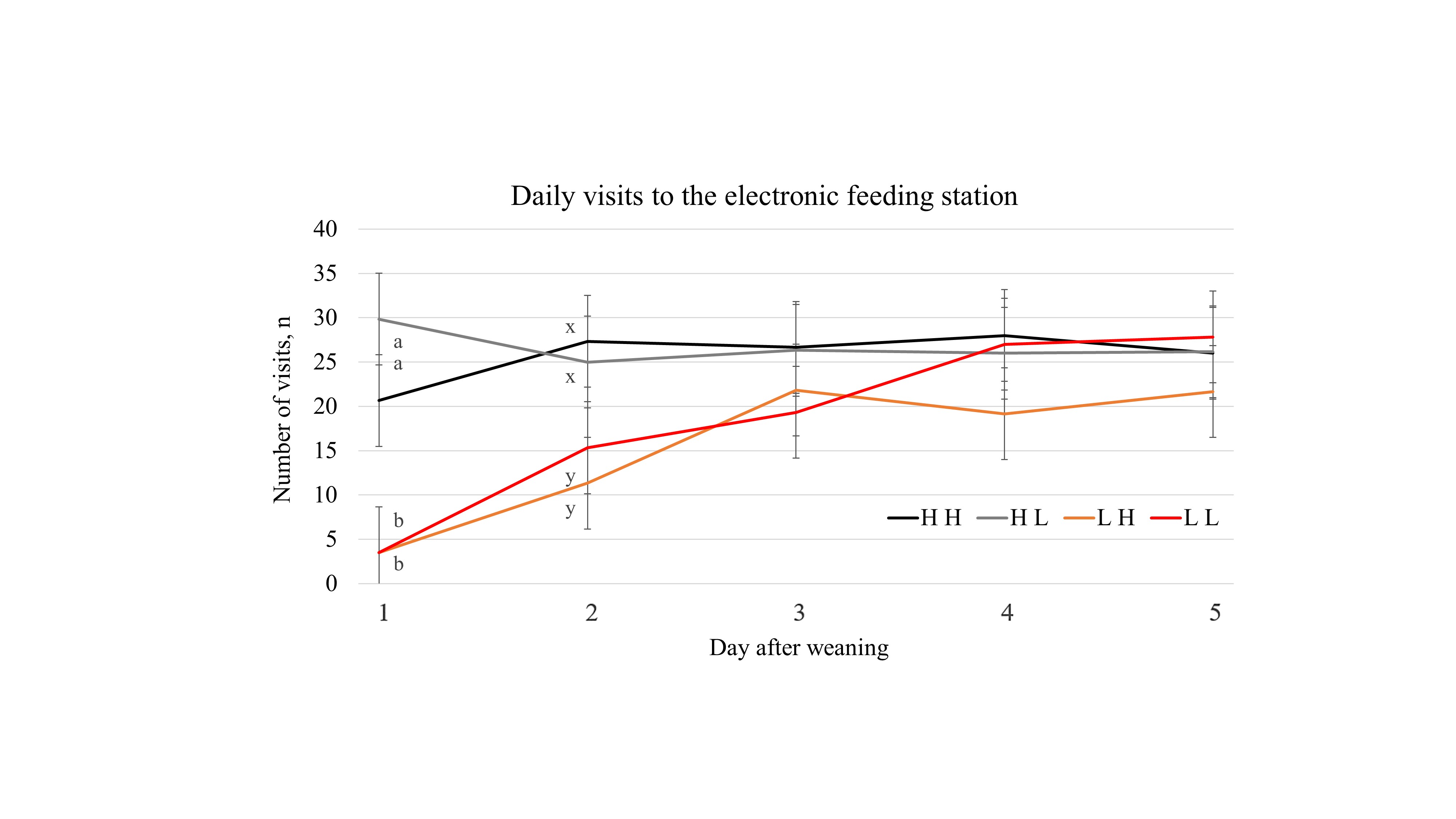

Supplement: skaf099_suppl_Supplementary_Materials [file skaf099_suppl_supplementary_materials.zip › Figure S1.jpg]

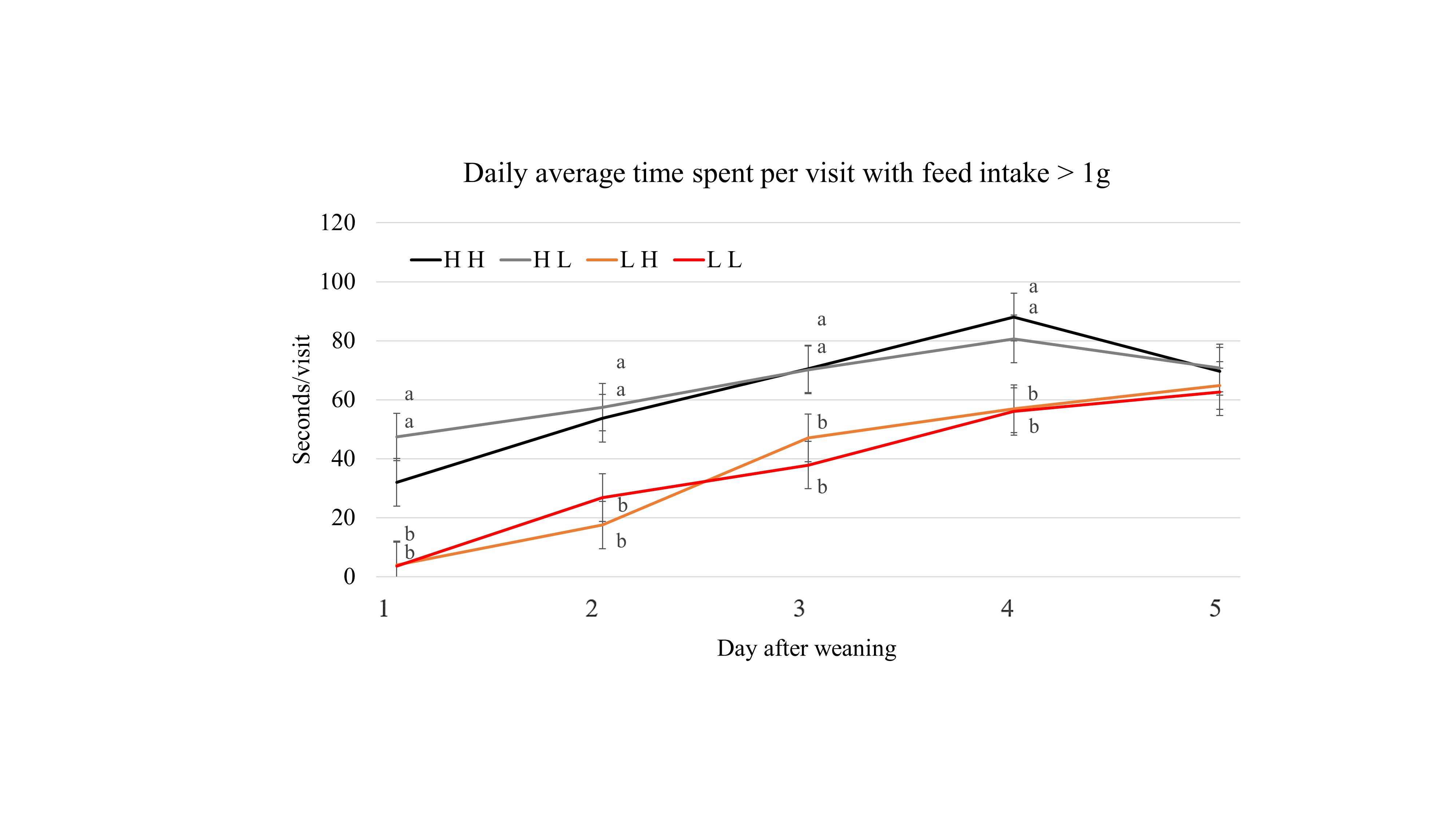

Supplement: skaf099_suppl_Supplementary_Materials [file skaf099_suppl_supplementary_materials.zip › Figure S2.jpg]

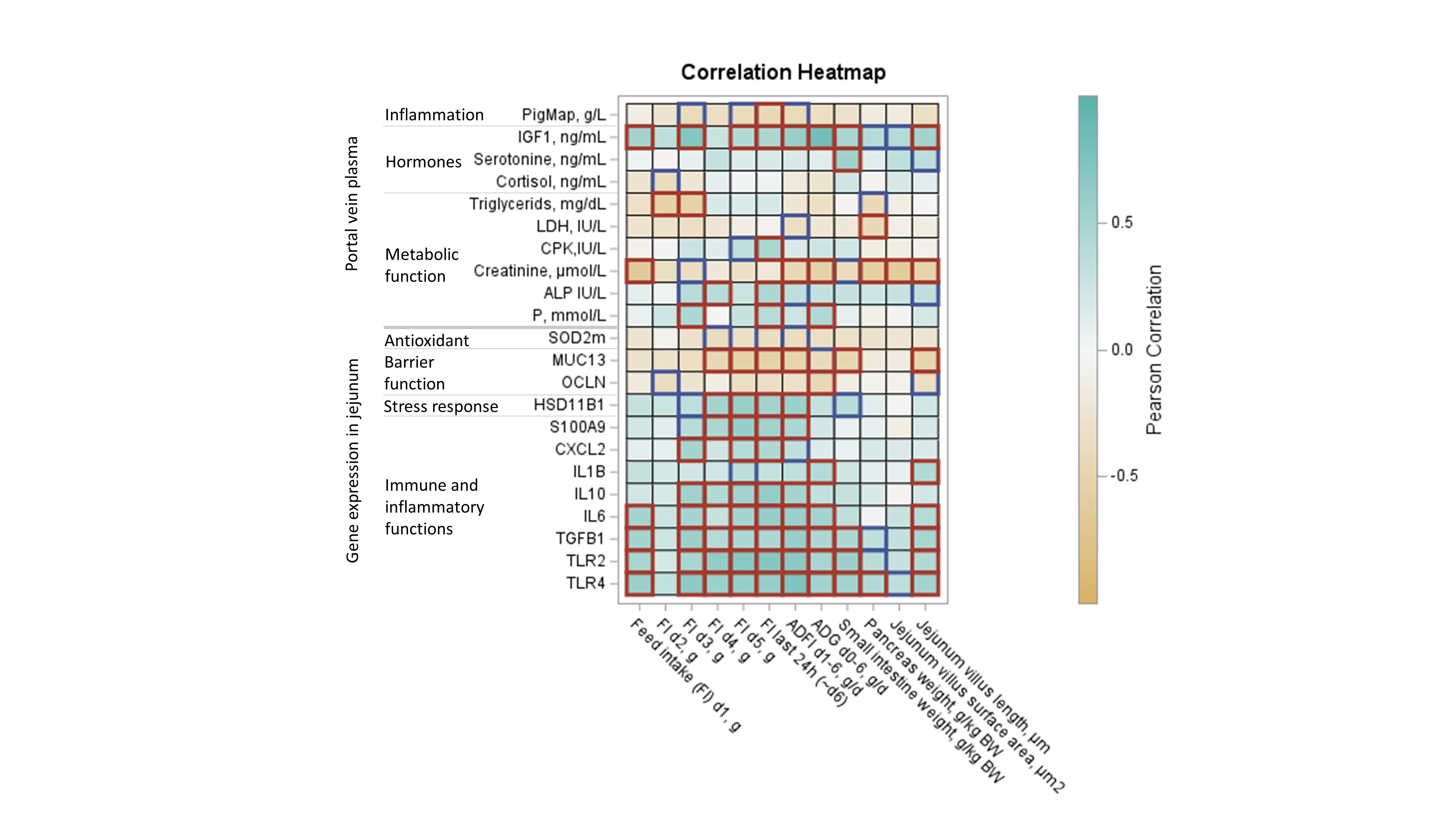

Supplement: skaf099_suppl_Supplementary_Materials [file skaf099_suppl_supplementary_materials.zip › Figure S3 Heatmap 2.jpg]
